# Impact of reboxetine plus oxybutynin treatment for obstructive sleep apnea on cardiovascular autonomic modulation

**DOI:** 10.1038/s41598-023-29436-9

**Published:** 2023-02-23

**Authors:** Elisa Perger, Paolo Castiglioni, Andrea Faini, Davide Soranna, Antonella Zambon, Debora Rosa, Stefano Vicini, Paolo Meriggi, Laura Pini, Claudia Baratto, Sergio Caravita, Ali Azarbarzin, Gianfranco Parati, Carolina Lombardi

**Affiliations:** 1 Sleep Disorders Center and Department of Cardiovascular, Neural and Metabolic Sciences, San Luca Hospital, IRCCS Istituto Auxologico Italiano, Milan, Italy; 2 IRCCS Fondazione Don Carlo Gnocchi, Milan, Italy; 3 Dipartimento di Biotecnologie e Scienze della Vita, Università degli Studi dell’Insubria, Varese, Italy; 4 Politecnico di Milano, Milan, Italy; 5 Biostatistics Unit, IRCCS Istituto Auxologico Italiano, Milan, Italy; 6 Department of Statistics and Quantitative Methods, University of Milano-Bicocca, Milan, Italy; 7 Respiratory Medicine Unit, ASST Spedali Civili di Brescia, Brescia, Italy; 8 Department of Clinical and Experimental Sciences, University of Brescia, Brescia, Italy; 9 Department of Management, Information and Production Engineering, University of Bergamo, Dalmine, BG Italy; 10 Brigham and Women’s Hospital and Harvard Medical School, Boston, MA USA; 11 Department of Medicine and Surgery, University of Milano-Bicocca, Milan, Italy

**Keywords:** Cardiology, Cardiovascular biology, Drug safety, Respiration

## Abstract

The combination of noradrenergic (reboxetine) plus antimuscarinic (oxybutynin) drugs (reb-oxy) reduced obstructive sleep apnea (OSA) severity but no data are available on its effects on cardiac autonomic modulation. We sought to evaluate the impact of 1-week reb-oxy treatment on cardiovascular autonomic control in OSA patients. OSA patients were randomized to a double-blind, crossover trial comparing 4 mg reboxetine plus 5 mg oxybutynin to a placebo for OSA treatment. Heart rate (HR) variability (HRV), ambulatory blood pressure (BP) monitoring (ABPM) over 24 h baseline and after treatment were performed. Baroreflex sensitivity was tested over beat-to-beat BP recordings. 16 subjects with (median [interquartile range]) age 57 [51–61] years and body mass index 30 [26–36]kg/m^2^ completed the study. The median nocturnal HR was 65 [60–69] bpm at baseline and increased to 69 [64–77] bpm on reb-oxy vs 66 [59–70] bpm on placebo (*p* = 0.02). The mean 24 h HR from ABPM was not different among treatment groups. Reb-oxy administration was not associated with any modification in HRV or BP. Reb-oxy increased the baroreflex sensitivity and did not induce orthostatic hypotension. In conclusion**,** administration of reb-oxy did not induce clinically relevant sympathetic overactivity over 1-week and, together with a reduction in OSA severity, it improved the baroreflex function.

## Introduction

Obstructive sleep apnea (OSA) is one of the most common sleep disorders affecting one-third of the population aged 30–70 years in Europe^1^. The repetitive collapse of the pharyngeal airway characteristic of OSA leads to intermittent oxygen desaturations and sleep fragmentation with downstream consequences, including an increased risk of cardiovascular disease, daytime sleepiness, and sympathetic nervous system over-activity^2,3^. Intermittent hypoxemia and arousals from sleep activate the sympathetic nervous system, being them the major contributors to the blood pressure (BP) and heart rate (HR) elevation, together with the release of inflammatory mediators^2,4^. Thus, untreated OSA may lead to cardiovascular, neurocognitive, metabolic, and daytime functional consequences over time, resulting in increased morbidity and mortality^5^.

In the last 40 years, the gold standard for OSA treatment has been nocturnal ventilation through continuous positive airway pressure (CPAP), which acts to pneumatically splint the pharynx open. If regularly employed, CPAP therapy might reduce cardiovascular risk and overall mortality rate^6,7^, partially by attenuating sympathetic over-activity^8,9^ and improving arterial baroreflex sensitivity, depressed by OSA^10–12^. Although OSA is effectively alleviated by this treatment, CPAP is poorly tolerated by many patients, with a consequent very low adherence^13,14^. Hence, there is an urgent need for alternative interventions that are safe and well-tolerated^15–17^.

Reduced pharyngeal dilator muscle tone and responsiveness during sleep are key contributors to OSA pathogenesis^18,19^. Responsible for the latter is a loss of noradrenergic drive and muscarinic inhibition to the upper airway muscles^20,21^. In the last years, these findings have stimulated research on pharmacological solutions aimed at improving neural control of pharyngeal muscle tone. In such a context, pharmacotherapy for OSA is moving its first steps with promising results provided by single-night studies^17,22^. We have recently performed a study aimed at expanding the knowledge on these pharmacological possibilities by conducting a randomized controlled trial to evaluate for the first time the effectiveness of the combination of reboxetine (a noradrenaline reuptake inhibitor agent) and oxybutynin (an antimuscarinic compound), administered over a 1-week period, versus placebo on patients with moderate-to-severe OSA with promising results^23^.

As known, noradrenaline is the main neurotransmitter of the sympathetic nervous system and muscarinic receptors are responsible for the vagal activity. Thus, a pending issue, not yet explored, is the possibility that the administration of noradrenergic reboxetine together with antimuscarinic oxybutynin might negatively affect the autonomic control of circulation^24,25^. On the other hand, the substantial decrease of the apnea-hypopnea index (AHI) with the resulting improvement of nocturnal hypoxemia induced by reb-oxy, might be also associated with beneficial effects on sympathetic over-activity, thus counterbalancing the direct sympathetic stimulation of these noradrenergic and antimuscarinic drugs.

The sympathetic and parasympathetic cardiovascular modulation can be assessed with direct measurements, or thanks to non-invasive reliable tools such as BP by 24 h ambulatory monitoring (ABPM), HR variability (HRV), and baroreceptor sensitivity (BRS)^24,26–28^.

In order to assess the effect of reboxetine plus oxybutynin (reb-oxy) on cardiovascular autonomic modulation in OSA patients, we performed 24 h ABPM and estimated BRS and HRV after 1-week of treatment in the frame of our randomized controlled cross-over trial. The outcomes were evaluated as the change from baseline during the week of reb-oxy administration and compared to placebo.

## Methods

### Patients and study design

We performed a randomized, double-blind, placebo-controlled, cross-over, single-center study of the combination of reb-oxy in adults with OSA documented by polysomnography (PSG). Participants were enrolled from July 2020 to October 2020 through our sleep clinic (Istituto Auxologico Italiano, Milan, Italy) and the trial ended when the previously calculated sample size of 16 patients was reached. Participants were randomized (1:1 ratio) to first receive a 4 mg reboxetine plus 5 mg oxybutynin or a matching placebo, and then to switch to the other treatment arm after a washout of 7–10 days. Study participants matching eligibility criteria performed a one-night inpatient PSG test, which served as the baseline measure for AHI. Subjects started taking the study drug the day after the baseline PSG, immediately before bedtime, and continued for 7 days in total. On the final dosing night of each week, participants performed in-lab PSG. The study was approved by the Ethics Committee on 18/02/2020 (approval n. 2020_02_18_03) and by the Italian drug agency AIFA (Agenzia Italiana del Farmaco) on 22/04/2020 (EudraCT n 2019-004917-15) and all methods were performed in accordance with the guidelines and regulations. Informed consent in writing was obtained from all study participants. The study was first registered at ClinicalTrials.gov (NCT04449133) on 26/06/2020.

### Measurements

#### Overnight in-lab polysomnography

PSG recording and data interpretation was performed in accordance with the American Academy of Sleep Medicine (AASM) scoring manual^29^. All studies were scored by the same specialized sleep clinician, blinded to treatment assignment, according to AASM scoring criteria^30^.

#### Heart rate variability

The beat-by-beat series of R-R intervals were extracted from 1 ECG channel of each PSG through a derivative-and-threshold algorithm, selecting a segment of at least 10-min duration without respiratory events in the supine position during non-REM stage 2 (N2) sleep for the HRV analysis. Premature beats were visually identified and manually removed, obtaining series of normal-to-normal intervals (NNI). Time-domain indexes of HRV were the root-mean-square of successive differences (RMSSD) and the percentage of beats with NNI value at least 50 ms longer or shorter than NNI of their preceding beat (pNN50)^28^. For the frequency-domain analysis, the NNI series was resampled evenly at 5 Hz linearly interpolating possibly missing beats. The Welch periodogram was estimated using 50% overlapped Hanning windows of 120 s length. The powers in the very-low-frequency (VLF, between 0.0025 and 0.04 Hz), low-frequency (LF, between 0.04 and 0.15 Hz), and high-frequency (HF, between 0.15 and 0.40 Hz) bands, as well as the LF/HF powers ratio, were obtained by integrating the periodogram^28^. The breathing rate was estimated from the fluctuations of QRS-complex amplitude of the ECG reflecting the respiratory movements of the thorax^31^. The series of the R peaks resampled at 5 Hz were high-pass filtered at 0.05 Hz to remove oscillations too long to be generated by respiratory movements; the Welch periodogram was calculated and the breathing rate measured as the frequency of the highest spectral peak.

#### Ambulatory blood pressure monitoring

ABPM was performed using a validated oscillometric device (TM2430; A&D Medical, Japan) to evaluate blood pressure (BP) changes for 24 h. Blood pressure was evaluated every 20 min during the day and every 30 min during the night. Day and night sub-periods were defined according to the personal logbook. 24 h, daytime and night-time average Systolic BP (SBP), Diastolic BP (DBP) and heart rate (HR), day and night SBP and DBP standard deviations (SD) were quantified to assess BP variability, SBP and DBP nocturnal falls, night/day ratios, average morning (7–11 a.m.) values and morning surge (defined as the difference between the lowest SBP or DBP value before the morning rise and the highest SBP or DBP value after awakening). The ABPM monitoring was assessed in the pre-screening visit at least 2 days before baseline PSG and between the 2nd and the 5th night of the two weeks of treatment to avoid disturbing the PSG night sleep.

#### Baroreflex function

ECG and non-invasive BP were measured by a Nexfin^®^ device (BMEYE, Amsterdam, The Netherlands). Patients underwent measurements at rest, without coffee intake in the previous 3 h and at the same time of the day before the PSG. Recordings were performed in a silent room without speaking and without any disturbing elements for 10 min while patients were lying supine, and then for 10 min while they were standing. The beat-by-beat values of SBP and DBP, derived from the continuous BP, recordings, and NNI from the ECG lead, were interpolated evenly at 5 Hz for spectral analysis. The sensitivity of the baroreflex control of heart rate (BRS) was estimated by the sequence technique^32^ (BRS_SEQ_), the transfer function technique (H_LF_)^33^, and the spectral method (α_LF_)^34^. As to BRS_SEQ_, the beat-to-beat series were scanned in search of sequences of 3 or more consecutive heartbeats in which a progressive SBP increase was followed, with a lag of zero, one or two beats, by a progressive NNI lengthening or, vice versa, in which a progressive SBP reduction was followed by a progressive NNI shortening. The slope of the regression line between SBP and NNI values in each sequence was taken as a local BRS estimate. The local estimates were averaged over the supine and standing periods separately to obtain the final BRS_SEQ_ values. As to the spectral and transfer function estimates, the SBP and NNI power spectra, and SBP-NNI cross-spectrum and coherency spectrum were calculated from the evenly resampled series at 5 Hz using 50% overlapped Hann data windows of 120s length. SBP and NNI powers were calculated over the LF band and the root square of the SBP/ NNI powers ratio evaluated only for spectral lines with squared coherence modulus > 0.3 provided the α_LF_ estimate of BRS. The ratio between the SBP-NNI cross-spectrum and NNI spectrum was calculated considering again only spectral lines with squared coherence modulus > 0.3 and averaged over the LF band, providing the H_LF_ estimate of BRS.

The DBP power spectrum was similarly estimated by the Welch periodogram. The baroreflex resonance was quantified by integrating the DBP and SBP power spectra over the LF band (DBP_LF_ and SBP_LF_). These indexes measure the power of the 10-s oscillations in arterial BP, which are considered a surrogate measure of sympathetic activity^35^.

For each index, the difference (delta Δ) between supine position and standing was also calculated.

### Statistical analysis

Categorical data were summarized by proportions, while continuous data by median [interquartile range] values. Percent changes in study variables from baseline after 1-week of placebo and after 1-week of reb-oxy were compared as continuous variables by using a two-tailed Wilcoxon matched-pairs signed-rank test. The effect of the drug combination on the cardiovascular autonomic nervous system reported here was a prespecified exploratory outcome, while the primary outcome was AHI change as reported in our previous publication^23^. According to study design, a sample size of 16 subjects was powered to detect an AHI reduction with Reb-Oxy than placebo (alpha 5%, power 80%), plus a ≈ 20% dropout{Perger, 2022 #223}.

A *p*-value of <0·05 was considered statistically significant. Statistical analyses were performed using Statistical Analysis Software (SAS Cary, NC, USA).

## Results

The anthropometric and clinical characteristics of the 16 participants are presented in Table 1. None had beta-blockers as chronic therapy. No serious adverse events occurred. The main sleep and OSA severity metrics are summarized in Table 2. Other results of this trial have been previously published^23^.

**Table 1:** Characteristics of the enrolled population (total number of subjects = 16).

General characteristics	
Age, years	57 [51–61]
Male	14 (88%)
Body mass index Kg/m^2^	30 [26–36]
Current smokers	8 (50%)
Risk factors and comorbidities	
Hypertension	7 (44%)
Diabetes	1 (6%)
Dyslipidemia	7 (44%)
Hypothyroidism	3 (19%)
Rheumatoid arthritis	1 (6%)
Pharmacological treatments	
ACE-I/ARB	6 (35%)
Calcium channel blocker	1 (6%)
Diuretics	1 (6%)
Antilipidemics	4 (25%)
Antidiabetics	1 (6%)
Antithrombotics	2 (13%)

ACE-I = angiotensin-converting enzyme inhibitor; ARB = angiotensin receptor blocker; Data are presented as absolute number (%) or median [interquartile range].

**Table 2:** Obstructive Sleep Apnea severity parameters (some reported from previous study^23^), main sleep characteristics, and heart rate variability (HRV) at baseline, on placebo, and on active drugs combination (n = 16); HRV was assessed from EKG of nocturnal PSG during N2 sleep at baseline, on placebo, and on active drugs combination.

	Baseline	Placebo	Reb-Oxy	*p*-value
Full night PSG				
AHI total, events/h	48.7 [34.8–56.6]	38.7 [29.0–47.8]	18.0 [12.5–21.4]	< 0.01
Hypoxic burden, %min/h	90.8 [69.5–154]	75.5 [68.1–168.0]	39.7 [25.4–55.3]	< 0.01
ODI 3%, events/h	42.7 [32.3–53.0]	36.8 [23.8–43.2]	31.4 [19.1–37.7]	0.03
Total sleep time, min	329.5 [301.0–368.8]	323.5 [274.4–351.4]	321.8 [283.0–362.9]	0.50
PLM index, events/h	0.0 [0.0–2.8]	0.0 [0.0–2.8]	0.5 [0.0–2.8]	0.81
HR, bpm	65 [59.5–69]	65.6 [58.8–69.55]	69.35 [63.8–76.75]	0.02
HRV in N2 phase				
RMSSD, ms^2^	40.4 [17.5–51.6]	24.7 [17.6–43.1]	32.4 [25.4–51.6]	0.38
pNN50	0.18 [0.01–0.32]	0.02 [0.001–0.18]	0.10 [0.03–0.35]	0.40
HF, ms^2^	300.0 [90.7–748.4]	189.7 [85.6–527.2]	328.8 [181.5–887.9]	0.53
LF, ms^2^	370.2 [136.9–1021.4]	222.8 [159.1–1099.7]	371.47 [227.8–614.94]	0.38
LF/HF	2.4 [0.9–3.9]	1.3 [0.9–2.4]	1.1 [0.6–1.7]	0.46
VLF, ms^2^	680.6 [341.1–2879.1]	504.4 [232.3–2095.3]	659.4 [434.4–885.6]	0.25

reb–oxy = reboxetine plus oxybutynin; AHI = apnea–hypopnea index; ODI = oxygen desaturation index; PLM = periodic legs movements; HR = heart rate; SD = standard deviation; RMSSD = Root Mean Square of the Successive Differences; pNN50 = the proportion of number of pairs of successive NN (R-R) intervals that differ by more than 50 ms; HF = high frequency; LF = low frequency; VLF = very low frequency.

Data are presented as median [interquartile] % changes are expressed as the median of percentage change. *P* values compare placebo versus reb-oxy.

*P*-values are calculated by comparing the percentage changes from baseline observed in the placebo versus reb-oxy.

### Heart rate and blood pressure

Median nocturnal heart rate (HR) during the PSG increased from 65 [60–69] bpm at baseline to 69 [64–77] bpm on reb-oxy and to 66 [59–70] bpm on placebo (*p*=0.02 for reb-oxy versus placebo) (Table 2). However, 24 h HR measured by ABPM was not significantly different between treatment groups, as shown in Table 3.

**Table 3:** Ambulatory blood pressure data at baseline, on placebo, and on reb-oxy drug combination (n = 16).

	Baseline	Placebo	Reb-Oxy	*p*-value
BP values				
24 h DBP, mmHg	83.0 [76.3–87.8]	83.0 [75.3–85.7]	79.6 [75.6–89.5]	0.68
24 h SBP**,** mmHg	131.6 [122.1–140.3]	129.5 [121.0–133.1]	121.5 [115.4–140.9]	0.72
24 h HR, bpm	78.0 [71.7–90.4]	80.2 [71.5–87.6]	79.1 [69–86.6]	0.60
Day-time DBP, mmHg	86.8 [82.2–92.6]	85.78 [80.4–88.3]	82.18 [78.6–94.7]	0.86
Day-time SBP, mmHg	138.7 [129.9–145.1]	133.0 [125.1–138.1]	125.0 [117.3–144.3]	0.46
Night-time DBP, mmHg	71.8 [65.9–76.0]	70.7 [67.1–76.5]	69 [65.6–76.5]	0.50
Night-Time SBP, mmHg	112.3 [106.1–124.2]	118.5 [108.6–126.2]	110.1 [107.6–132.3]	0.28
BP variability				
Morning surge DBP, mmHg	20.0 [15.5–40.0]	15.0 [6.5–34.5]	20.0 [10.0–33.0]	0.60
Morning surge SBP, mmHg	48.0 [31.5–87.5]	33.0 [23.5–45.5]	32.0 [25.5–39.5]	0.38
Day-time DBP SD mmHg	18.1 [11.5–20.6]	15.7 [12.7–18.3]	15.7 [11.7–19.1]	0.90
Day-time SBP SD mmHg	17.9 [13.9–24.9]	16.9 [12.2–21.1]	17.2 [15.7–19.8]	0.79
Night-time DBP SD mmHg	9.9 [7.9–13.2]	10.6 [8.4–13.7]	9.6 [9.3–10.9]	0.23
Night-time SBP SD mmHg	12.1 [10.4–13.8]	13.2 [11.2–17.3]	12.3 [9.1–16.6]	0.08

reb–oxy = reboxetine plus oxybutynin; DBP = diastolic blood pressure; SBP = systolic blood pressure. Data are presented as median [interquartile range].

*P* values compare placebo versus reb-oxy.

*P*-values are calculated by comparing the percent changes from baseline observed after placebo versus reb-oxy.

Reb-oxy did not significantly modify 24 h, daytime, and night-time DBP and SBP (Table 3). Morning surge was not increased on reb-oxy as compared to placebo and BP variability was not significantly different during the day and the night between groups.

Based on both time and frequency domain analyses, reb-oxy administration was not associated with any modifications in HRV (Table 2). The respiratory frequencies remained in the HF band in both groups.

### Baroreflex sensitivity and resonance

**Figure 1:**
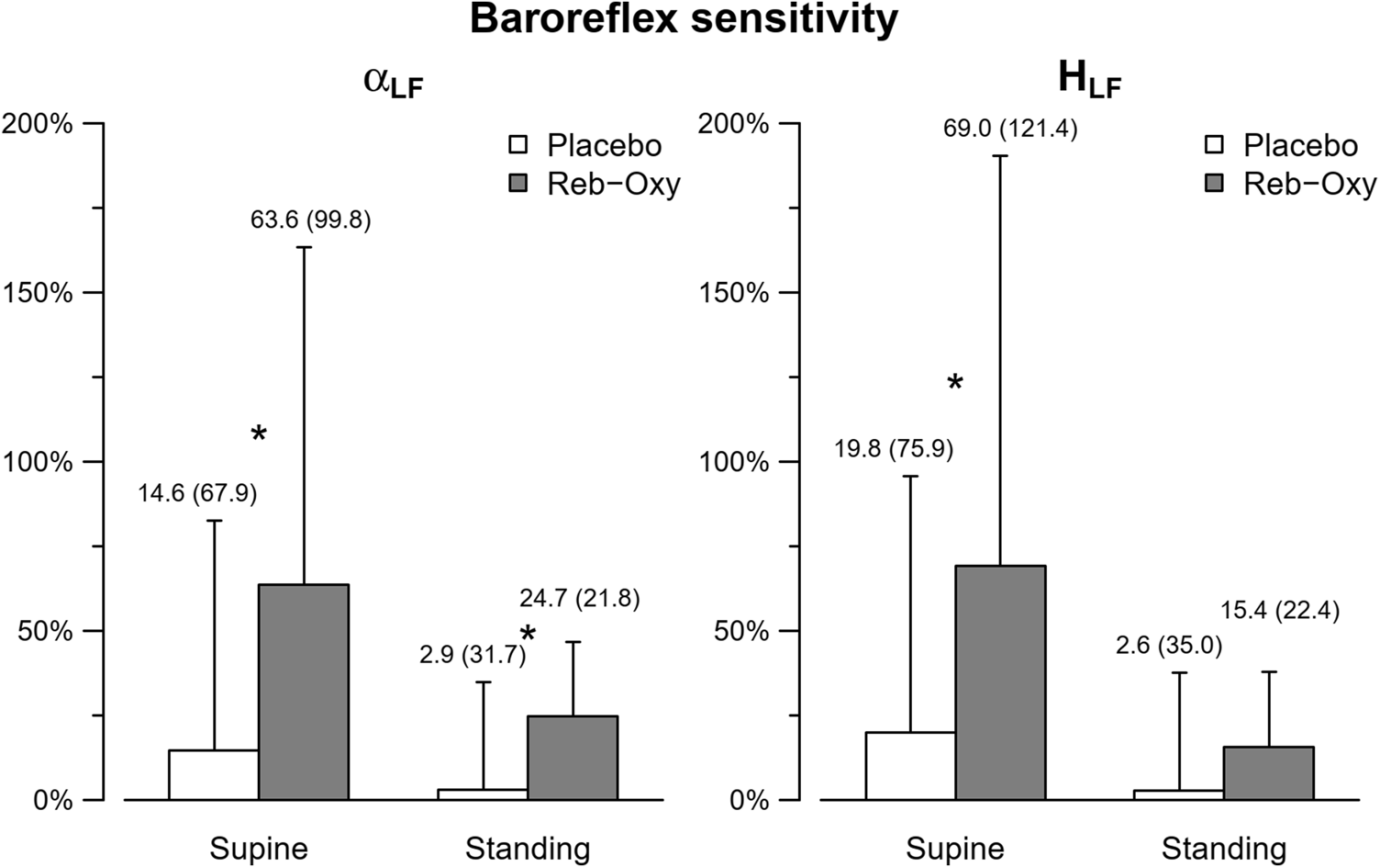
Baroreflex sensitivity by the spectral method. αLF percent change (left) and HLF percent change (right) from baseline under placebo (white bar) and reb-oxy (grey bar) in supine and standing positions. Data are shown as medians (median absolute deviation); the * indicates statistical significance between reb-oxy and placebo. See Table 4 for the original values.

**Figure 2:**
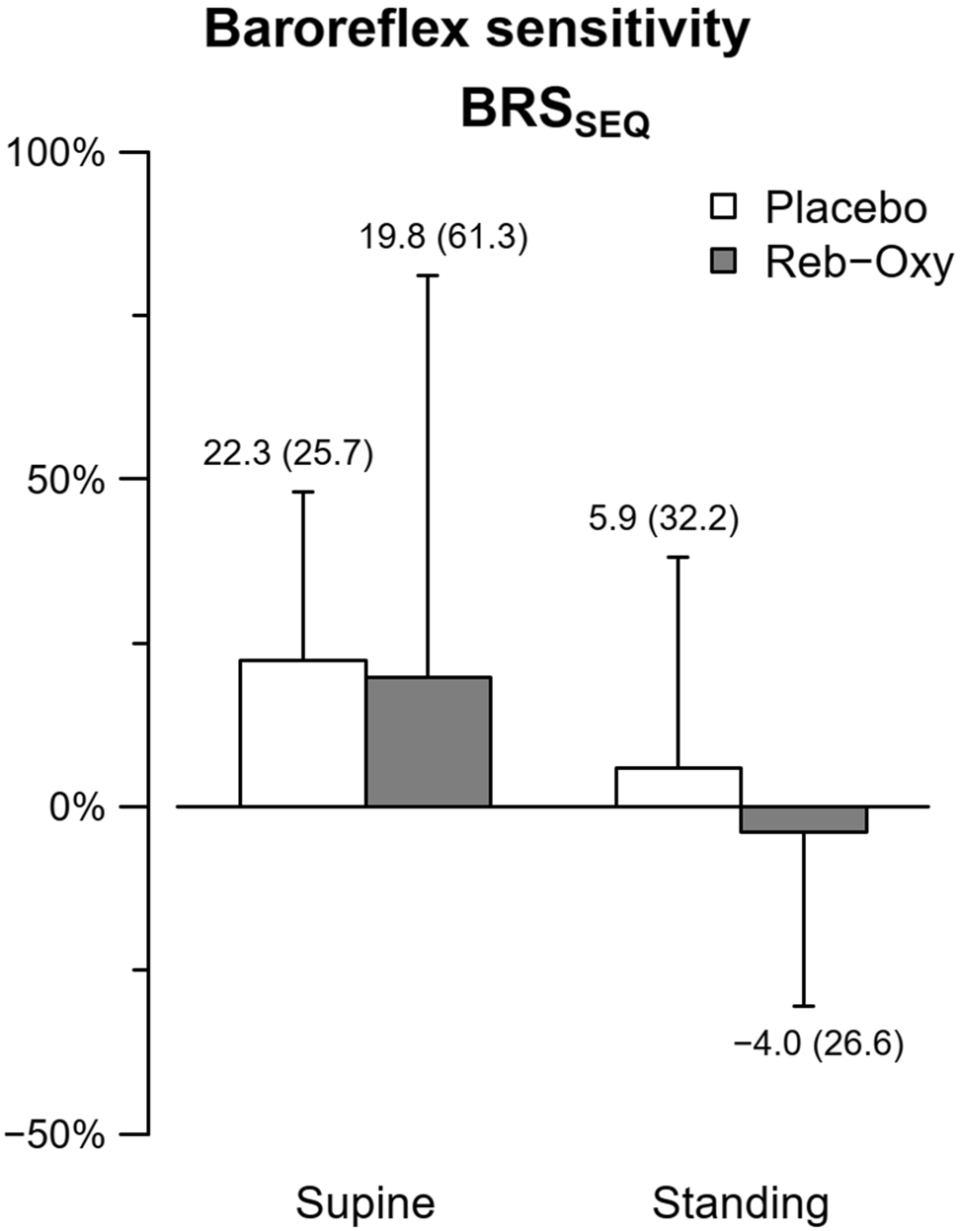
Baroreflex sensitivity by the sequence method. BRS_SEQ_ percent change from baseline under placebo (white bar) and reb-oxy (grey bar) in supine and standing positions. Data are shown as medians (standard error). See Table 4 for the original values. BRS_SEQ_ percent change from baseline under placebo (white bar) and reb-oxy (grey bar) in supine and standing positions. Data are shown as medians (median absolute deviation).

Table 4 reports the estimates of baroreflex sensitivity and the measures of baroreflex resonance in BP spectral powers at baseline, after 1 week of placebo, and after 1 week of reb-oxy treatment. In the supine position, both the spectral and the transfer function estimates of baroreflex sensitivity were significantly greater after reb-oxy administration than on placebo. A similar effect was observed in standing position: both α_LF_ and H_LF_ increased more after treatment with reb-oxy than after the placebo treatment, the difference being significant for the spectral estimates. Figure 1 shows the corresponding percent changes from baseline recordings. The percent changes in α_LF_ from supine to standing were similar on placebo (+ 49% [+ 32 to + 60] %) and on reb-oxy (+4 6% [+ 33 to + 66] %, *p = *0.58). The percent changes in H_LF_ from supine to standing were similar on placebo (+ 49% [+ 30 to + 59] %) and on reb-oxy (+ 47% [+ 39 to + 69] %, *p* 0.42). When considering changes from baseline to treatment, no differences between placebo and reb-oxy were found for BRS_SEQ_ (Table 1 and Figure 2). The percent changes in BRS_SEQ_ from supine to standing were also similar between placebo (+ 36% [+ 26 to + 53] %) and reb-oxy (+ 54% [+ 34 to + 71] %, *p* 0.13).

**Table 4:** Baroreflex sensitivity and resonance at baseline, on placebo, and on reb-oxy (n = 16).

	Baseline	Placebo	Reb-Oxy	*p*-value
BRS ms/mmHg				
α_LF_ supine	5.3 [3.8–8.6]	6.0 [4.2–8.8]	9.3 [7.8–10.8]	0.02
α_LF_ standing	2.6 [2.0–4.6]	3.1 [2.0–4.3]	3.7 [2.5–6.8]	0.03
H_LF_ supine	3.9 [2.8–6.6]	5.1 [3.5–6.8]	8.2 [5.8–10.5]	0.01
H_LF_ standing	2.0 [1.6–4.0]	2.2 [1.5–3.3]	3.1 [1.9–4.8]	0.07
**BRS**_**SEQ**_ supine	6.7 [4.2–7.6]	7.2 [3.3–9]	9.3 [8–12.6]	0.29
**BRS**_**SEQ**_ standing	3.9 [2.3–5.3]	3.4 [3.2–4.5]	3.9 [2.4–5]	0.08
Baroreflex resonance, mmHg^2^				
** SBP**_**LF**_ supine	7.8 [3.9–14.2]	9.3 [4.7–11.7]	2.8 [2.1–3.8]	< 0.01
** SBP**_**LF**_ standing	14.6 [11.2–20.4]	17.0 [10.9–21.4]	5.8 [4.2–9.8]	< 0.01
** DBP**_**LF**_ supine	2.7 [1.9–5.3]	3.5 [1.8–5.8]	1.3 [1.1–2.0]	< 0.01
** DBP**_**LF**_ standing	5.0 [3.8–9.2]	5.9 [4.1–9.4]	2.4 [1.7–3.4]	< 0.01

BRS: baroreflex sensitivity; SBP = systolic blood pressure; DBP = diastolic blood pressure; LF = low frequency; HLF = BRS estimation by transfer function technique in LF band and α_LF_ = BRS estimation by spectral method in LF band (for further details, see methods). Data are expressed as percentage or median [interquartile].

*P* values compare placebo versus reb-oxy.

*P*-values are calculated by comparing the percent changes from baseline observed after placebo versus reb-oxy.

**Figure 3:**
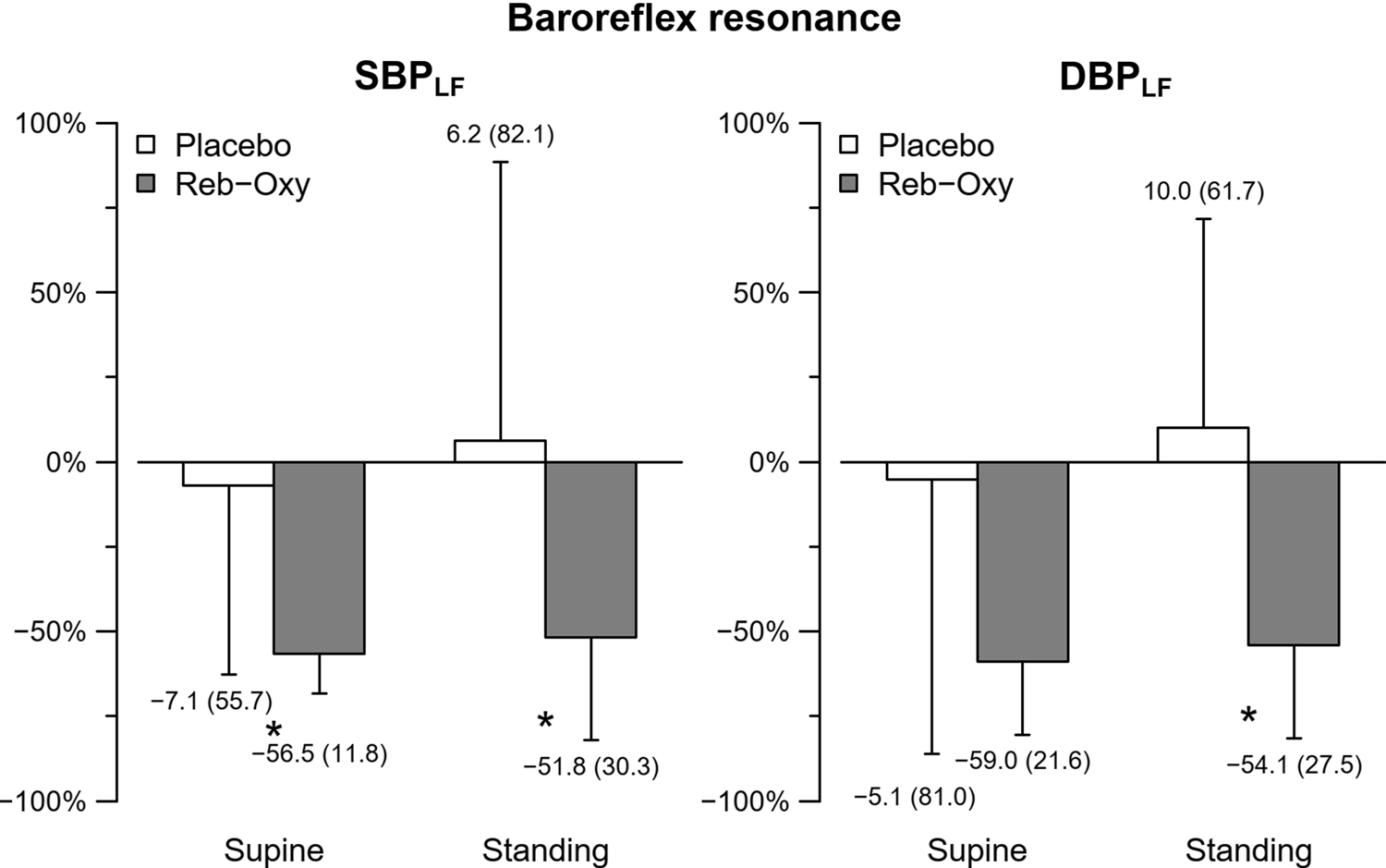
Baroreflex resonance. Percent changes of spectral powers in the low-frequency band for systolic (SBP_LF_, *left*) and diastolic (DBP_LF_, *right*) blood pressure from baseline in placebo (white bar) and reb-oxy (grey bar) in the supine and standing positions separately: data as medians (median absolute deviation). See Table 4 for the original values. The symbol * indicates statistical significance between reb-oxy and placebo.

Regarding the baroreflex resonance in BP spectral powers, SBP_LF_ and DBP_LF_ were significantly and substantially lower after reb-oxy treatment than on placebo in both postures (Table 1 and Figure 3). The percent changes in SBP_LF_ from supine to standing were similar on placebo (− 86% [− 204 to − 28]) and on reb-oxy (− 108.1% [− 266 to − 35], *p* = 0.60). This was the case also for the percent changes in DBP_LF_ from supine to standing (placebo: − 101% [− 153 to − 31] %; reb-oxy: − 72% [− 101 to − 43] %, *p* 0.52).

Reb-oxy did not induce orthostatic hypotension nor any modification of parasympathetic activity indices. The respiratory frequencies remained in the HF band in all treatment groups.

## Discussion

We recently demonstrated that one-week bedtime administration of reboxetine plus oxybutynin reduces OSA severity^23^. Here, we add that, in OSA patients, this treatment is not associated with short-term autonomic dysregulation. Particularly, during indirect evaluations, the tested drug combination does not induce either sympathetic overactivity or orthostatic hypotension as it could be expected by the reboxetine mechanism of action.

Our results show that one-week administration of reboxetine plus oxybutynin: (i) did not increase BP during the day or the night; (ii) did not enhance cardiac sympathetic modulation as reflected by nighttime HRV; (iii) did not impair but rather increased the sensitivity of the baroreflex control of heart rate; and (iv) decreased the baroreflex resonance in BP spectra, a surrogate measure for the vascular sympathetic modulation. To date, this is the first evaluation of the impact of the new OSA pharmacologic therapy on cardiovascular autonomic regulation.

Reboxetine is highly selective for norepinephrine transporters and has a low affinity with muscarinic, histamine-H1, and adrenergic α1 receptors^36^. Reboxetine is prescribed for major depressive diseases, dysthymia, and attention-deficit/hyperactivity disorder, its antidepressant effects being related to the sustained increase in norepinephrine levels in the central nervous system^37^. As a noradrenergic drug, reboxetine would be expected to increase sympathetic activity. In fact, in previous studies on healthy volunteers, 4 mg of reboxetine has been associated with an increase in HR^38,39^. Oxybutynin is an anticholinergic drug used for bladder over-activity. Its effect on muscarinic cardiac receptors determines inhibition of vagal activity with an increase in HR^40^. HR is indeed primarily modulated by the opposing autonomic influences of parasympathetic and sympathetic activity. Thus, theoretically, the administration of this drug combination would be expected to inhibit cardiac parasympathetic stimulation together with a direct sympathomimetic effect.

### Reb-oxy and HR/BP levels in OSA

A previous pharmacological trial for OSA treatment showed that a single-night administration of atomoxetine plus oxybutynin in OSA patients led to a small (2.6 bpm) overnight increase in HR^22^. Similarly, in our study, HR during the night only slightly increased (by 3.7 bpm) on reb-oxy versus placebo during PSG. Moreover, the 24 h HR evaluated during the ABPM was similar in the two groups. Previous studies in healthy subjects reported an increase in SBP and DBP after reboxetine administration^38,39,41^, whereas neutral effects on BP have been reported in long-term treatment with reboxetine^37,42,43^. In our patients, BP did not differ between placebo and reb-oxy and we did not observe orthostatic hypotension after 1 week of reb-oxy administration, confirming the neutral effect previously reported even in a population of OSA patients. Morning surge and BP variability, additional markers of sympathetic activity, did not increase during reb-oxy administration, which, again, represents a reassuring clinical result considering the administration of a noradrenergic drug.

### Reb-oxy and HRV in OSA

Using HRV analysis, OSA patients were shown to be characterized by a predominance of sympathetic markers and by a reduction in parasympathetic indices^44–46^. This is in line with the demonstration that sympathetic overactivity is a key mediator of adverse cardiovascular consequences in OSA^47^. In fact, autonomic derangement and sympathovagal imbalance are associated with arrhythmias, which are often observed in patients with OSA, contributing to their cardiovascular mortality^48^. Previous studies showed that reboxetine influences the autonomic nervous system by reducing the HF power of HRV in healthy volunteers^39^. Oxybutynin, in turn, showed an anti-cholinergic effect according to HRV indexes^40^. By contrast, our study points out that in OSA patients one week of reb-oxy did not increase sympathetic activity, as shown by no changes in the HRV spectral index of sympathovagal balance, i.e., the LF/HF power ratio, and had no significant influence on HRV indexes of parasympathetic cardiac control: RMSSD, pNN50, and the HF power.

### Reb-oxy and arterial baroreflex cardiovascular modulation in OSA

An unbalanced cardiac baroreflex modulation has been shown to favor cardiovascular complications and arrhythmias. Particularly, in OSA patients the known reduction in BRS during both wake and sleep is associated with the development of arterial hypertension and with excessive daytime sleepiness^24,49^. Our study does not report any worsening of BRS after reb-oxy treatment, as it could be theoretically expected considering the autonomic effects of the noradrenergic reboxetine and anticholinergic oxybutynin. On the contrary, our data show a significantly improved sensitivity of arterial baroreflex modulation of HR, in particular in the LF band (α_LF_ and H_LF_). It has been shown that α_LF_ and H_LF_ reflect baroreflex modulation of HR through both the sympathetic and the parasympathetic neural influences. Conversely, BRS_SEQ_ mainly reflects the vagal HR modulation^50^. Thus, our findings of increased α_LF_ and H_LF_ accompanied by no changes in BRS_SEQ_ suggest that one-week treatment with reb-oxy improved BRS mainly through a reduction of cardiac sympathetic modulation, with no evident changes in vagal control of HR.

Moreover, our study demonstrates that reb-oxy largely decreased the power of SBP and DBP oscillations at LF, which have been suggested to be an expression of the resonance in the baroreflex loop at a frequency of 0.1 Hz, considered a surrogate measure for sympathetic vascular regulation. Thus, when considering all the above findings, our study suggests the occurrence of a reduction in the overactivity of sympathetic cardiovascular control induced by sleep apneas in OSA patients after one week of reb-oxy treatment. This reduction, however, was not accompanied by major alterations in posture-induced changes in cardiovascular control, as demonstrated by the lack of orthostatic hypotension and other signs of autonomic imbalance.

The improvement in baroreflex cardiac modulation we observed during wake is likely to represent a positive consequence of nocturnal apneas reduction, as previously reported after long-term OSA treatment with CPAP^12^. Thus, similarly to what happens with CPAP treatment, it might be supposed that the beneficial effect of drug combination on the severity of OSA would have mitigated the sympathetic hyperactivity induced by OSA events^9,12,51^. The reduction of the hypoxic burden, together with a reduced frequency of arousals from sleep determined by reb-oxy, might have determined the beneficial effect on the apnea-hypopnea-related sympathetic overflow^52,53^.

## Limitations

Even if the present study offers unique data on the effects of a novel drug treatment for OSA over the longest administration period tested so far, we acknowledge a few limitations. First, our findings on the short-term impact of Reb-Oxy on cardiac autonomic modulation in OSA patients cannot be generalize to other populations and need to be confirmed over a longer period. Second, the relatively small sample size was powered to detect significant changes in AHI, not in baroreflex or HRV indices, possibly precluding the detection of smaller effects of the treatment. Third, by study design we evaluated cardiovascular autonomic modulations only by indirect measures, as direct measures of sympathetic activity such as norepinephrine spillover or muscle sympathetic nerve traffic were excluded.

## Conclusions

We demonstrate for the first time that the combination of the noradrenergic drug reboxetine and the anticholinergic drug oxybutynin could safely be administrated in OSA patients, who are typically characterized by sympathetic overactivity due to sleep apnea *per se*. Being efficacious on OSA severity, these drugs could also positively act to improve the deranged cardiovascular autonomic modulation typical of OSA. Future intervention trials, including a larger number of OSA patients followed over longer treatment periods are needed to assess whether these favorable clinical and pathophysiological effects are also associated with a reduction in cardiovascular morbidity and mortality.

## Supplementary Information

Supplementary Information.

## Data Availability

Data used for this study are available from the corresponding author upon reasonable request.
