# Supplementary material for: Impact of reboxetine plus oxybutynin treatment for obstructive sleep apnea on cardiovascular autonomic modulation
Source: Sci Rep. 2023 Feb 23;13:3178. doi: 10.1038/s41598-023-29436-9 (PMC9950422; doi:10.1038/s41598-023-29436-9)
Supplement: Supplementary file 1 — Supplementary Information. [file 41598_2023_29436_MOESM1_ESM.docx]

**Impact of Reboxetine plus Oxybutynin treatment for Obstructive Sleep Apnea on Cardiovascular Autonomic Modulation**

Elisa Perger^1, 2^, Paolo Castiglioni^3^, Andrea Faini^1^, Davide Soranna^4^, Antonella Zambon^4,5^, Debora Rosa^1^, Stefano Vicini^1, 2^, Paolo Meriggi^3^, Laura Pini^6,7^, Claudia Baratto^1^, Sergio Caravita^1,8^, Ali Azarbarzin^9^, Gianfranco Parati^1, 2*^, Carolina Lombardi^1, 2*^

From the ^1^Istituto Auxologico Italiano, IRCCS, Sleep Disorders Center & Department of Cardiovascular, Neural and Metabolic Sciences, San Luca Hospital, Milan, Italy; ^2^Department of Medicine and Surgery, University of Milano-Bicocca, Milan, Italy; ^3^IRCCS Fondazione Don Carlo Gnocchi, Milan, Italy; ^4^ IRCCS Istituto Auxologico Italiano, Biostatistics Unit, Milan, Italy; ^5^Department of Statistics and quantitative methods, University of Milano-Bicocca, Milan, Italy; ^6^ Respiratory Medicine Unit, ASST Spedali Civili di Brescia, Brescia, Italy; ^7^Department of Clinical and Experimental Sciences, University of Brescia, Brescia, Italy; ^8^Department of Management, Information and Production Engineering, University of Bergamo, Dalmine (BG); ^9^Division of Sleep and Circadian Disorders, ^9^Brigham and Women’s Hospital and Harvard Medical School, Boston, MA

* these authors contributed with the same effort to the project

**SUPPLEMENTAL FILE**

**Methods:**

***Measurements***

*Heart Rate Variability:* The beat-by-beat series of R-R intervals were extracted from 1 ECG channel of each PSG through a derivative-and-threshold algorithm, selecting a segment of at least 10-minute duration without respiratory events in the supine position during non-REM stage 2 (N2) sleep for the HRV analysis. Premature beats were visually identified and manually removed, obtaining series of normal-to-normal intervals (NNI). Time-domain indexes of HRV were the root-mean-square of successive differences (RMSSD) and the percentage of beats with NNI value at least 50 ms longer or shorter than NNI of their preceding beat (pNN50) (1). For the frequency-domain analysis, the NNI series was resampled evenly at 5 Hz linearly interpolating possibly missing beats. The Welch periodogram was estimated using 50% overlapped Hanning windows of 120 s length. The powers in the very-low-frequency (VLF, between 0.0025 and 0.04 Hz), low-frequency (LF, between 0.04 and 0.15 Hz), and high-frequency (HF, between 0.15 and 0.40 Hz) bands, as well as the LF/HF powers ratio, were obtained by integrating the periodogram (1). The breathing rate was estimated from the fluctuations of QRS-complex amplitude of the ECG reflecting the respiratory movements of the thorax (2). The series of the R peaks resampled at 5 Hz were high-pass filtered at 0.05 Hz to remove oscillations too long to be generated by respiratory movements; the Welch periodogram was calculated and the breathing rate measured as the frequency of the highest spectral peak.

*Ambulatory Blood Pressure Monitoring:* ABPM was performed using a validated oscillometric device (TM2430; A&D Medical, Japan) to evaluate blood pressure (BP) changes for 24 hours. Blood pressure was evaluated every 20 min during the day and every 30 min during the night. Day and night sub-periods were defined according to the personal logbook. 24h, daytime and night-time average Systolic BP (SBP), Diastolic BP (DBP) and heart rate (HR), day and night SBP and DBP standard deviations (SD) were quantified to assess BP variability, SBP and DBP nocturnal falls, night/day ratios, average morning (7-11 a.m.) values and morning surge (defined as the difference between the lowest SBP or DBP value before the morning rise and the highest SBP or DBP value after awakening). The ABPM monitoring was assessed in the pre-screening visit at least 2 days before baseline PSG and between the 2^nd^ and the 5^th^ night of the two weeks of treatment to avoid disturbing the PSG night sleep.

*Baroreflex Function:* ECG and non-invasive BP were measured by a Nexfin® device (BMEYE, Amsterdam, The Netherlands). Patients underwent measurements at rest, without coffee intake in the previous 3 hours and at the same time of the day before the PSG. Recordings were performed in a silent room without speaking and without any disturbing elements for 10 minutes while patients were lying supine, and then for 10 minutes while they were standing. The beat-by-beat values of SBP and DBP, derived from the continuous BP, recordings, and NNI from the ECG lead, were interpolated evenly at 5 Hz for spectral analysis. The sensitivity of the baroreflex control of heart rate (BRS) was estimated by the sequence technique (3) (BRS_SEQ_), the transfer function technique (H_LF_) (4), and the spectral method (α_LF_)(5). As to BRS_SEQ_, the beat-to-beat series were scanned in search of sequences of 3 or more consecutive heartbeats in which a progressive SBP increase was followed, with a lag of zero, one or two beats, by a progressive NNI lengthening or, vice versa, in which a progressive SBP reduction was followed by a progressive NNI shortening. The slope of the regression line between SBP and NNI values in each sequence was taken as a local BRS estimate. The local estimates were averaged over the supine and standing periods separately to obtain the final BRS_SEQ_ values. As to the spectral and transfer function estimates, the SBP and NNI power spectra, and SBP-NNI cross-spectrum and coherency spectrum were calculated from the evenly resampled series at 5 Hz using 50% overlapped Hann data windows of 120s length. SBP and NNI powers were calculated over the LF band and the root square of the SBP/ NNI powers ratio evaluated only for spectral lines with squared coherence modulus >0.3 provided the α_LF_ estimate of BRS. The ratio between the SBP-NNI cross-spectrum and NNI spectrum was calculated considering again only spectral lines with squared coherence modulus >0.3 and averaged over the LF band, providing the H_LF_ estimate of BRS.

The DBP power spectrum was similarly estimated by the Welch periodogram. The baroreflex resonance was quantified by integrating the DBP and SBP power spectra over the LF band (DBP_LF_ and SBP_LF_). These indexes measure the power of the 10-second oscillations in arterial BP, which are considered a surrogate measure of sympathetic activity (6).

For each index, the difference (delta Δ) between supine position and standing was also calculated.

**SUPPLEMENTAL REFERENCES**

1. Electrophysiology TFotESoCtNASoP. Heart Rate Variability. *Circulation* 1996; 93: 1043-1065.

2. Moody GB, Mark RG, Bump MA, Weinstein JS, Berman AD, Mietus JE, Goldberger AL. Clinical Validation of the ECG-Derived Respiration (EDR) Technique. 2008.

3. Parati G. Arterial baroreflex control of heart rate: determining factors and methods to assess its spontaneous modulation. *The Journal of Physiology* 2005; 565: 706-707.

4. Robbe HW, Mulder LJ, Rüddel H, Langewitz WA, Veldman JB, Mulder G. Assessment of baroreceptor reflex sensitivity by means of spectral analysis. *Hypertension* 1987; 10: 538-543.

5. Pagani M, Somers V, Furlan R, Dell'Orto S, Conway J, Baselli G, Cerutti S, Sleight P, Malliani A. Changes in autonomic regulation induced by physical training in mild hypertension. *Hypertension* 1988; 12: 600-610.

6. Julien C. The enigma of Mayer waves: Facts and models. *Cardiovasc Res* 2006; 70: 12-21.
